# Quantitative assessment of plantar pressure patterns in relation to foot deformities in people with hereditary motor and sensory neuropathies

**DOI:** 10.1186/s12984-023-01172-1

**Published:** 2023-05-16

**Authors:** Bente E. Bloks, Lise M. Wilders, Jan Willem K. Louwerens, Alexander C. Geurts, Jorik Nonnekes, Noël L.W. Keijsers

**Affiliations:** 1 Department of Research, Sint Maartenskliniek, Nijmegen, The Netherlands; 2 Department of Rehabilitation, Radboud University Medical Center, Donders Institute for Brain, Cognition and Behaviour, Nijmegen, The Netherlands; 3 Department of Rehabilitation, Sint Maartenskliniek, Nijmegen, The Netherlands; 4 Department of Orthopedics, Sint Maartenskliniek, Nijmegen, The Netherlands; 5 Department of Sensorimotor Neuroscience, Donders Institute for Brain, Cognition and Behaviour, Radboud University, Nijmegen, The Netherlands

**Keywords:** Hereditary motor and sensory neuropathies, Charcot-Marie-Tooth, Plantar pressure, Foot deformity

## Abstract

**Background:**

Hereditary motor and sensory neuropathies (HMSN), also known as Charcot-Marie-Tooth disease, are characterized by affected peripheral nerves. This often results in foot deformities that can be classified into four categories: (1) plantar flexed first metatarsal, neutral hindfoot, (2) plantar flexed first metatarsal, correctable hindfoot varus, (3) plantar flexed first metatarsal, uncorrectable hindfoot varus, and (4) hindfoot valgus. To improve management and for the evaluation of surgical interventions, a quantitative evaluation of foot function is required. The first aim of this study was to provide insight into plantar pressure of people with HMSN in relation to foot deformities. The second aim was to propose a quantitative outcome measure for the evaluation of surgical interventions based on plantar pressure.

**Methods:**

In this historic cohort study, plantar pressure measurements of 52 people with HMSN and 586 healthy controls were evaluated. In addition to the evaluation of complete plantar pressure patterns, root mean square deviations (RMSD) of plantar pressure patterns from the mean plantar pressure pattern of healthy controls were calculated as a measure of abnormality. Furthermore, center of pressure trajectories were calculated to investigate temporal characteristics. Additionally, plantar pressure ratios of the lateral foot, toes, first metatarsal head, second/third metatarsal heads, fifth metatarsal head, and midfoot were calculated to measure overloading of foot areas.

**Results:**

Larger RMSD values were found for all foot deformity categories compared to healthy controls (p < 0.001). Evaluation of the complete plantar pressure patterns revealed differences in plantar pressure between people with HMSN and healthy controls underneath the rearfoot, lateral foot, and second/third metatarsal heads. Center of pressure trajectories differed between people with HMSN and healthy controls in the medio-lateral and anterior-posterior direction. The plantar pressure ratios, and especially the fifth metatarsal head pressure ratio, differed between healthy controls and people with HMSN (p < 0.05) and between the four foot deformity categories (p < 0.05).

**Conclusions:**

Spatially and temporally distinct plantar pressure patterns were found for the four foot deformity categories in people with HMSN. We suggest to consider the RMSD in combination with the fifth metatarsal head pressure ratio as outcome measures for the evaluation of surgical interventions in people with HMSN.

## Background

Hereditary motor and sensory neuropathies (HMSN), also known as Charcot-Marie-Tooth disease, is a group of progressive diseases affecting motor and sensory peripheral nerves [1]. All types of HMSN are characterized by distal muscle weakness and somatosensory impairments [2]. As a result of the distal muscle weakness, foot deformities are common and develop over time. These foot deformities are heterogenous but can be classified into four categories. The first category comprises a passively correctable plantar flexed position of the first metatarsal (MT1), which is usually the first change in foot position in people with HMSN [3]. The plantar flexed position of MT1 may become more prominent and structural over time, forcing the hindfoot into a varus position during loading. In this second category, the hindfoot is still passively correctable to a neutral position [3]. In the third category, it has become impossible to passively correct the hindfoot to a neutral position, resulting in an uncorrectable varus deformity. The fourth category, pes planovalgus, is less common in people with HMSN and typically follows from a normal foot in cases of rapid progression of muscle weakness [3].

In most people, foot deformities result in gait and balance impairments and, especially in people with varus deformity of the hindfoot, pain and pressure sores [1]. Therefore, adequate management of foot impairments is crucial. Such management primarily includes conservative treatment options (mainly orthopedic footwear), but, in the case of varus deformity of the hindfoot, surgical treatment options can be considered as well [4]. To enable adequate treatment, foot impairments should be evaluated accurately and thoroughly, especially when different surgical treatment modalities are considered. Clinical assessment typically focuses on the examination of the foot position in loaded and unloaded conditions and assessment of passive range of motion (ROM) of the ankle-foot joints. However, this is mainly focused on the static foot position and passive functioning of the foot, whereas the functional manifestations of foot impairments during gait are of great clinical interest. Therefore, to improve management, there is a need for quantitative evaluation of foot function in people with HMSN. Moreover, a quantitative assessment of foot function is crucial for the scientific evaluation of surgical interventions.

One way to evaluate foot function during walking is by measuring plantar pressure patterns. In people with HMSN, a decreased contact surface area and increased mean pressures, peak pressures, and pressure-time integrals have been found [5–7]. However, previous research has been inconclusive regarding the foot regions under which the pressures and pressure-time integrals are increased. This might be due to the variation in foot deformities that were included in previous studies without clearly distinguishing between them [5–7] since plantar pressure distributions are directly related to foot deformities [8]. For a better clinical and scientific evaluation of foot function and for the evaluation of surgical interventions, plantar pressure patterns should, therefore, be investigated for all four foot deformity categories separately.

Previous research divided the foot into a fixed number of anatomical regions, often called “masks”, to study the plantar pressure of different foot regions. However, definitions of these foot regions were inconsistent among studies [5–7], which makes it difficult to compare their results. Furthermore, applying a masking method results in loss of information on the complete plantar pressure pattern, comprising the plantar pressure distributions within the masks. Moreover, no quantitative assessment has been done on the temporal aspect of plantar pressure in people with HMSN in relation to foot deformities. Therefore, in addition to masking methods, there is a need for a more detailed investigation in a spatial and temporal way of plantar pressure in people with HMSN taking into account the different foot deformity categories.

The aims of this study were (1) to provide insight into plantar pressure patterns of people with HMSN in relation to the four foot deformity categories and (2) to propose a quantitative outcome measure based on plantar pressure patterns that could be used for the scientific evaluation of surgical interventions in people with foot deformities caused by HMSN. People with HMSN and the four foot deformity categories were included in this study. Regarding the first aim, complete plantar pressure patterns, root mean square deviations from the mean plantar pressure pattern of healthy controls, center of pressure trajectories, and plantar pressure ratios for different foot areas were calculated for the four foot deformity categories. Based on these results and the capacity of the calculated outcome measures to discriminate between the four foot deformity categories, a quantitative outcome measure is proposed for the scientific evaluation of surgical interventions. The proposed outcome measure should especially be focused on foot deformities with a varus position of the hindfoot since surgical interventions are considered in that case [4]. We expected to find increased plantar pressure underneath the head of MT1 in the case of a plantar flexed position of the first ray. Additionally, increased plantar pressure underneath the lateral side of the foot was expected in individuals with a varus deformity of the hindfoot, most prominently in the case of an uncorrectable varus deformity. Increased plantar pressure underneath the medial side of the foot was expected in individuals with a valgus deformity of the hindfoot.

## Methods

### Participants and design

In this historic cohort study, people with HMSN were included who had visited the outpatient clinic of the gait expertise center at the Sint Maartenskliniek and who had undergone an instrumented gait analysis including plantar pressure measurements between February 2018 and September 2021. Female individuals older than 11 years and male subjects older than 13 years were included to ensure sufficient skeletal maturity [9]. Plantar pressure patterns of both feet were measured. However, each foot was analyzed separately, because of differences in foot deformity between both feet. In total, 194 feet of 97 people with HMSN were screened for inclusion. Out of the 194 feet, 110 feet were excluded because of previous ankle/foot surgery (n = 78), a lack of three successful plantar pressure measurements (n = 13), incomplete footprints due to severe equinus deformity that made identification of foot regions impossible (n = 7), the age of the subject at the time of measurement (n = 4), a previous ankle/foot fracture (n = 3), other ankle/foot problems unrelated to HMSN (n = 2), the absence of foot deformities as determined by clinical examination (n = 2), or a lack of good pictures that made clinical examination impossible (n = 1). This resulted in 84 feet of 52 people that were included in the analysis. Additionally, plantar pressure patterns from a group of healthy controls, consisting of 586 people without disabilities that affect their walking pattern (including foot deformities), were available as a reference. This data set of healthy controls has previously been described [10] and was expanded by plantar pressure data of 174 people. Plantar pressure patterns of both feet of the healthy controls were used. Table 1 presents the subject characteristics. The type of HMSN was derived from the electronic patient record of the Sint Maartenskliniek.

**Table 1:** Subject characteristics

Group		n (people/feet)	Sex (% women)	Age (years, mean ± SD)	Type of HMSN (type I/type II/unknown)
Healthy controls		586/1172	50.2	48.6 ± 13.4	-
Total HMSN group		52/84	40.4	42.7 ± 17.1	(41/9/2)
	Plantar flexed first metatarsal with neutral hindfoot	19/24	42.1	41.2 ± 16.2	(14/4/1)
	Plantar flexed first metatarsal with correctable hindfoot varus	29/39	37.9	40.0 ± 16.2	(24/4/1)
	Plantar flexed first metatarsal with uncorrectable hindfoot varus	8/9	12.5	49.6 ± 11.2	(6/2/0)
	Hindfoot valgus	10/12	60.0	41.1 ± 22.2	(8/1/1)

### Examinations

Clinical examination took place to assess foot posture and flexibility to identify the foot deformity category. During the outpatient clinic at the Sint Maartenskliniek, frontal and sagittal plane pictures of both feet were taken in loaded and unloaded conditions from distances between approximately 30 centimeters and 1 meter. These pictures were independently assessed by two raters. First, the position of the first metatarsal bone relative to the other metatarsal bones was classified as either “plantar flexed” or “neutral”. This was done based on the sagittal plane pictures in unloaded condition, as this makes it possible to identify a plantar flexed position of the first metatarsal when this position is still passively correctable. Second, the hindfoot position in double leg stance was assessed based on the posterior frontal plane pictures and was classified as either “neutral”, “varus”, or “valgus”. When the raters differed in their judgement, they discussed the pictures together to reach consensus. The flexibility of the hindfoot was evaluated by two physiotherapists during the outpatient clinic at the Sint Maartenskliniek. To assess the passive ROM of the calcaneus, people were lying in prone position with an extended position of the hip and a flexed position of the knee. The ROM of the calcaneus was assessed by placing the point of rotation of a goniometer posterior on the ankle in between the medial and lateral malleolus. The proximal part of the goniometer was placed on the posterior midline of the lower leg and the distal part of the goniometer was placed on the posterior midline of the calcaneus [11]. If the calcaneus could reach an angle of zero degrees with respect to the lower leg, the flexibility of the hindfoot was classified as “correctable”. If not, the flexibility of the hindfoot was classified as “uncorrectable”. Thereafter, all feet were classified into one of the four HMSN subgroups: (1) plantar flexed first metatarsal with neutral position of the hindfoot during loading (“neutral”), (2) plantar flexed first metatarsal with passively correctable hindfoot varus during loading (“correctable varus”), (3) plantar flexed first metatarsal with passively uncorrectable hindfoot varus during loading (“uncorrectable varus”), and (4) hindfoot valgus position during loading (“valgus”).

A Footscan pressure plate (RSscan, Olen, Belgium) was used to record the plantar pressure data at 500 Hz. This 0.5 m long pressure plate consisted of 4096 sensors with dimensions of 7.5 × 5 mm. The pressure plate was placed on top of a force plate (Kistler, Winterthur, Switzerland) and synchronized with this force plate using the RSscan 3D-box. Data were collected during barefoot walking at a comfortable, self-selected walking speed. During each walking trial, subjects had to step on the pressure plate with one foot. Three to five successful walking trials were performed for both the left and the right foot.

### Data analyses

All plantar pressure patterns were rescaled to a standard foot and corrected for foot progression angle according to a previously described method [12]. Foot length and width were obtained by calculating the distance between respectively the most posterior part of the heel and the forefoot line and between the most medial and most lateral part of the forefoot. To obtain the foot progression angle, the mean angle of the tangent lines to the medial and lateral side of the foot was computed. To normalize the plantar pressure pattern, the size was rescaled based on the foot length and width. Furthermore, the plantar pressure pattern was rotated through the foot progression angle. As a result of this normalization, plantar pressure patterns of different subjects could be compared, and the plantar pressures could be analyzed at pixel level. Plantar pressure patterns of each foot were averaged over the three to five performed walking trials. To reduce the influence of body weight and walking velocity, the individual pixel pressures were normalized for total pressure by dividing the pressure of each pixel by the sum of the pressures of all pixels [10]. Subsequently, the individual pixel pressures were multiplied by the mean total pressure of all subjects to make it easier to interpret the resulting pressure values as they represent typical pressure values.

**Fig. 1:**
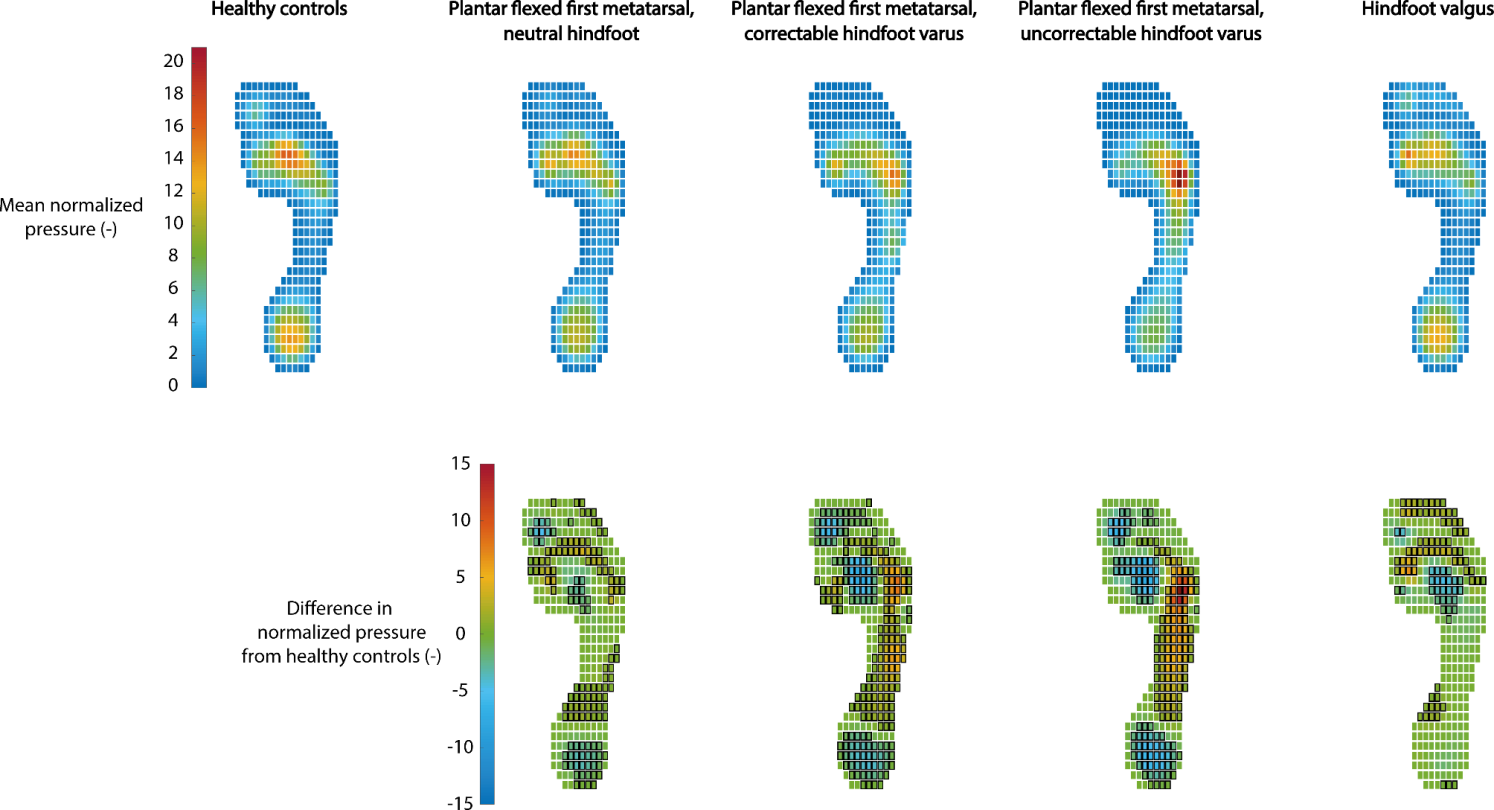
Normalized plantar pressure patterns. The top panels show the mean normalized plantar pressure patterns of healthy controls and the four HMSN subgroups. The lower panels show the differences in normalized plantar pressure from healthy controls for each HMSN subgroup. Positive values (yellow/red colors) indicate increased pressure, whereas negative values (blue colors) indicate decreased pressure compared to healthy controls. Pixels with a black border indicate a statistically significant difference (p < 0.01)

**Fig. 2:**
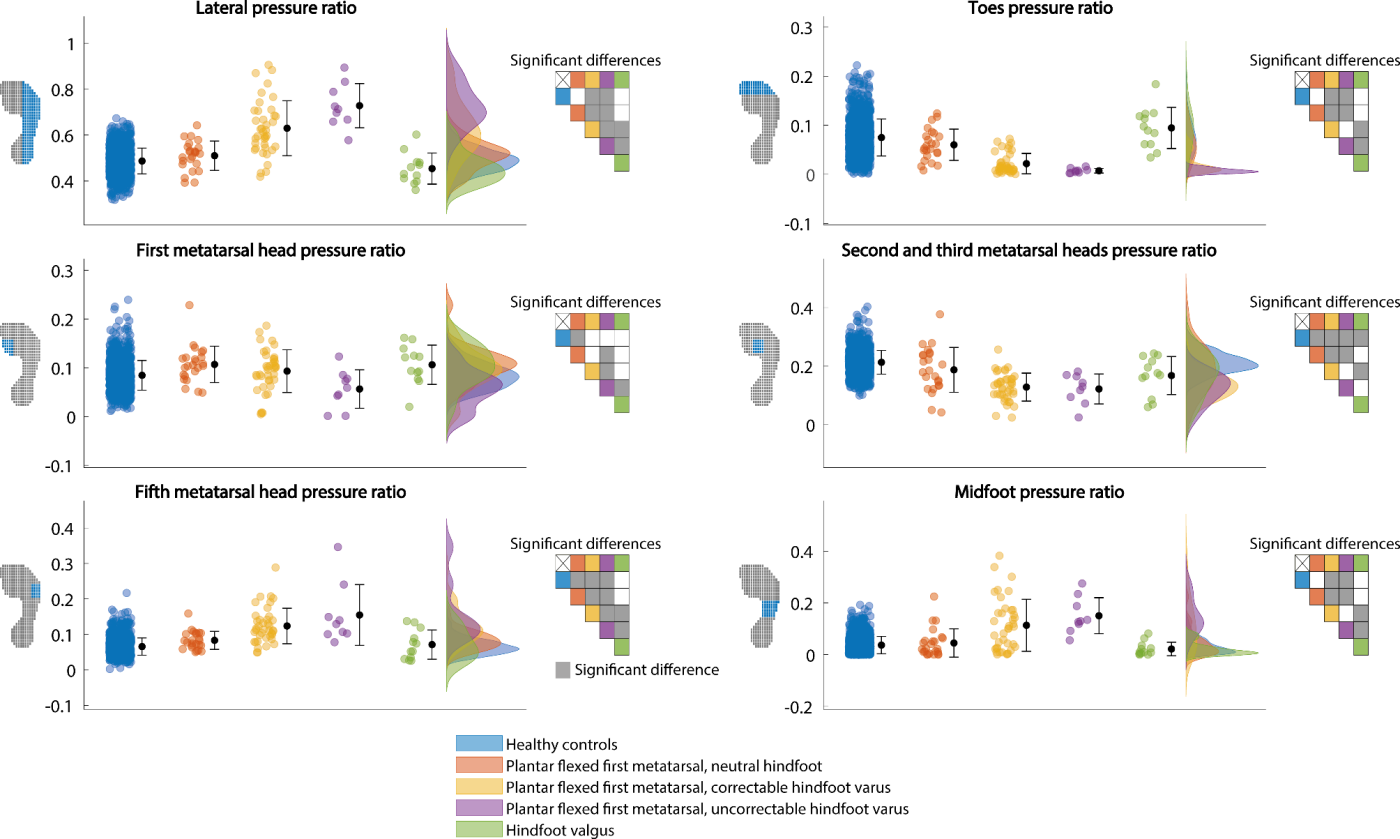
Plantar pressure ratios. Average plantar pressure ratios and standard deviations are depicted in black. The individual data points and distributions are depicted in the colors corresponding to the (sub)groups. On the left side of each graph, the definition of the pressure region for which the pressure ratio was calculated is depicted. On the right side of each graph, the statistically significant differences between (sub)groups are depicted. A grey box indicates a significant difference and a white box indicates no significant difference between the two (sub)groups on the corresponding row and column of a box, indicated by the colors

Root mean square deviations (RMSD) of the plantar pressure patterns from the mean plantar pressure pattern of healthy controls were calculated as a measure of abnormality by evaluating the differences in plantar pressure at pixel level. The upper left panel of Fig. 1 shows the mean plantar pressure pattern of healthy controls. The RMSD was also calculated for healthy controls to identify the variation within this reference group. Center of pressure trajectories in the medio-lateral and anterior-posterior directions were calculated and normalized to plantar pressure length and width to investigate the temporal aspect of plantar pressure. Furthermore, plantar pressure ratios of the lateral foot, toes, first metatarsal head, second and third metatarsal heads, fifth metatarsal head, and midfoot relative to the whole foot were calculated to measure whether a certain foot area was overloaded. The foot areas were defined as percentages of normalized plantar pressure pattern width from medial to lateral and length from posterior to anterior: lateral foot (57–100% of width), toes (84–100% of length), first metatarsal head (61–80% of length and 7–33% of width), second/third metatarsal heads (64–80% of length and 34–61% of width), fifth metatarsal head (61–77% of length and 79–100% of width), and midfoot (38–57% of length and 57–100% of width). In Fig. 2, the definitions of the foot areas are depicted. As an indication of walking velocity [13], center of pressure velocities in anterior-posterior direction were calculated from the plantar pressure data.

### Statistical analyses

The RMSD values, plantar pressure ratios, and center of pressure velocities of all feet were compared between healthy controls and the HMSN subgroups by one-way analysis of variance (ANOVA) tests. Post hoc pairwise comparison Bonferroni tests were performed to identify significant differences between (sub)groups. Statistical significance was defined as p < 0.05.

To compare the individual plantar pressure patterns at pixel level between the HMSN subgroups and healthy controls, independent samples t-tests were performed. The p-values were corrected based on clustering of pixels with a similar deviation in plantar pressure from the healthy control values (i.e., increase or decrease). The number of clusters that were created in this way was used to correct the p-value for the number of comparisons. This method was derived from neuroscience [14] and previously used for the statistical evaluation of plantar pressure patterns [15]. As can be seen in the lower panels of Fig. 1, 5 clusters of pixels with a similar deviation from healthy controls can be identified (i.e. heel, midfoot/lateral foot, second/third metatarsal heads, first metatarsal head, and toes), which means that a p-value < 0.01 (0.05/number of clusters) was considered as statistically significant.

One-way ANOVA tests were performed to compare the center of pressure trajectories between (sub)groups by statistical parametric mapping [16]. Post hoc independent samples t-tests with Bonferroni correction were performed to identify differences between (sub)groups. Statistical significance was defined as p < 0.05.

## Results

### Root mean square deviations

RMSD values were significantly different between (sub)groups (F(4,1251) = 105.2, p < 0.001, Table 2). Post hoc tests revealed significant differences in RMSD values between all (sub)groups, except between the neutral subgroup and the valgus subgroup.

**Table 2:** Root mean square deviations (RMSD)

Group		Root mean square deviation (mean ± SD)
Healthy controls		1.93 ± 0.54
HMSN subgroups		
	Plantar flexed first metatarsal with neutral hindfoot	2.68 ± 0.85
	Plantar flexed first metatarsal with correctable hindfoot varus	3.44 ± 1.16
	Plantar flexed first metatarsal with uncorrectable hindfoot varus	4.32 ± 2.20
	Hindfoot valgus	2.80 ± 0.80

### Plantar pressure patterns

Figure 1 shows the mean normalized plantar pressure patterns of the HMSN subgroups and healthy controls as well as the differences in mean normalized plantar pressure patterns from healthy controls for each HMSN subgroup. For all HMSN subgroups, there was a significant decrease in normalized plantar pressure underneath the distal part of the second/third metatarsals compared to healthy controls (p < 0.01). Underneath the fifth metatarsal head and midfoot, a significant increase in normalized plantar pressure was found for the correctable varus and uncorrectable varus subgroups compared to healthy controls (p < 0.01). Furthermore, a significant decrease in normalized plantar pressure underneath the heel and hallux was found for the neutral subgroup and the correctable varus and uncorrectable varus subgroups compared to healthy controls (p < 0.01). For the valgus subgroup, a significant increase in normalized plantar pressure was found underneath the toes compared to healthy controls (p < 0.01).

### Plantar pressure ratios

Figure 2 shows the plantar pressure ratios. The mean lateral pressure ratio in healthy controls was 0.49 ± 0.06, and the mean pressure ratios of the toes, first metatarsal head, second and third metatarsal heads, fifth metatarsal head, and midfoot varied between 0.04 and 0.21. For all plantar pressure ratios, significant differences were found between (sub)groups (lateral: F(4,1251) = 90.8, p < 0.001, toes: F(4,1251) = 28.2, p < 0.001, first metatarsal: F(4,1251) = 6.9, p < 0.001, second and third metatarsal heads: F(4,1251) = 53.1, p < 0.001, fifth metatarsal head: F(4,1251) = 71.9, p < 0.001, midfoot: F(4,1251) = 57.9, p < 0.001). Post hoc tests showed increased lateral, fifth metatarsal head, and midfoot pressure ratios and a decreased toes pressure ratio for the correctable varus and uncorrectable varus subgroups compared to the other HMSN subgroups and healthy controls. The uncorrectable varus subgroup showed increased lateral and fifth metatarsal head pressure ratios and a decreased first metatarsal head pressure ratio compared to the correctable varus subgroup. First metatarsal head, second and third metatarsal heads, and fifth metatarsal head pressure ratios were significantly different between the neutral subgroup and healthy controls. For the valgus subgroup, the second and third metatarsal heads pressure ratio were significantly decreased compared to healthy controls.

### Center of pressure trajectories

**Fig. 3:**
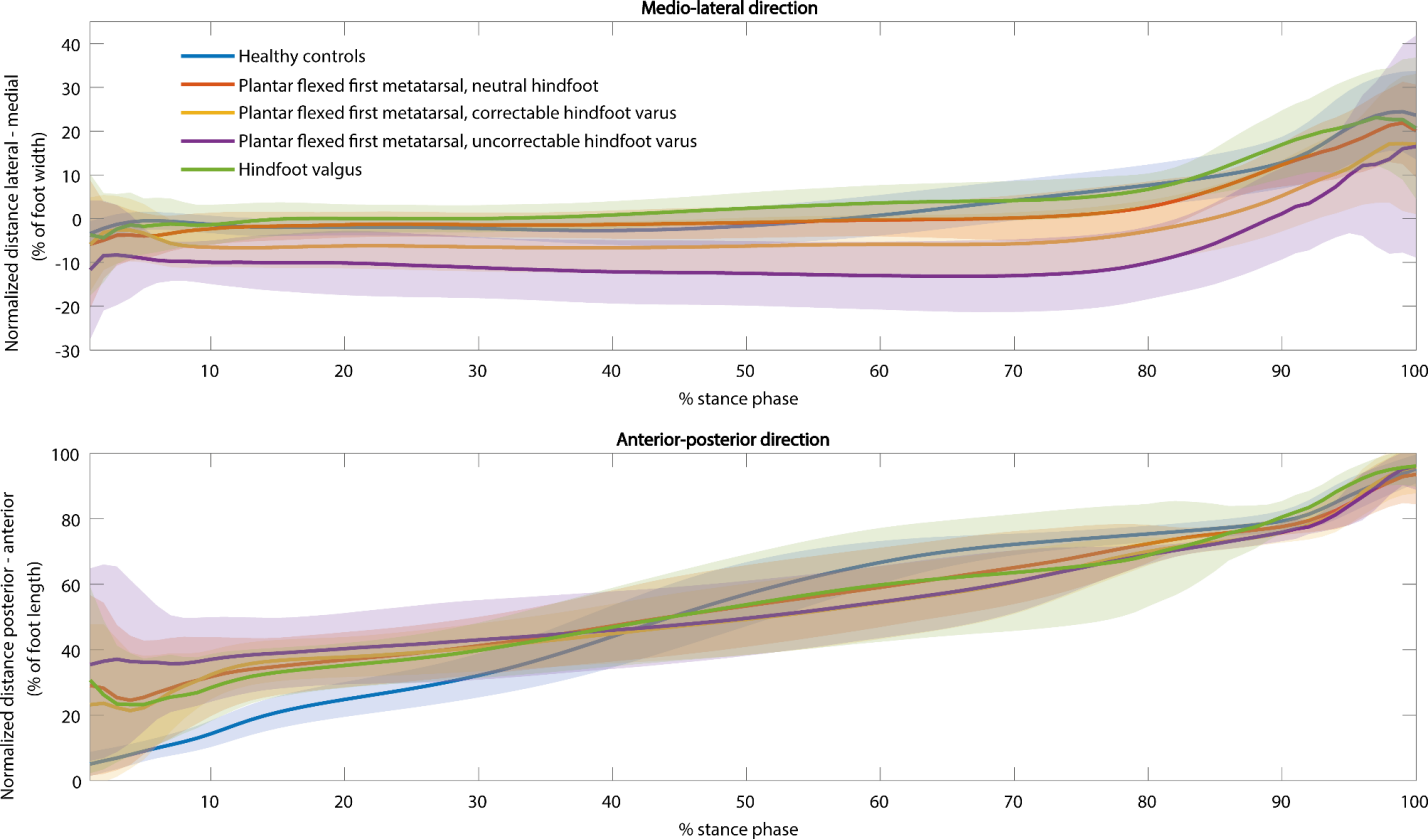
Center of pressure trajectories. In the upper panel, the mean center of pressure trajectory ± standard deviation in the medio-lateral direction over the stance phase is presented. The y-axis indicates the normalized distance from the midline of the foot, expressed as a percentage of foot width. In the lower panel, the mean center of pressure trajectory ± standard deviation in the anterior-posterior direction over the stance phase is presented. The y-axis indicates the normalized distance from the most posterior part of the heel, expressed as a percentage of foot length

Figure 3 shows the center of pressure trajectories for the medio-lateral and anterior-posterior directions. In the medio-lateral direction, the center of pressure trajectory was significantly different between (sub)groups during the entire stance phase (F(4,1251) = 3.9, p < 0.001). Post hoc tests revealed a significantly more lateral center of pressure trajectory for the neutral, correctable varus, and uncorrectable varus subgroups compared to healthy controls (neutral: between 1-9% and 68–84% of the stance phase, correctable varus: at 1% and between 3-100% of the stance phase, uncorrectable varus: between 1-96% and 96–98% of the stance phase). For the valgus subgroup, the center of pressure trajectory was significantly more medial compared to healthy controls (at 26% and between 32-45% of the stance phase). Furthermore, the center of pressure trajectory was significantly more lateral for the correctable varus subgroup compared to the neutral subgroup (between 10-47% and 84–90% of the stance phase), the uncorrectable varus subgroup compared to the neutral subgroup (between 7-90% of the stance phase), the correctable varus subgroup compared to the valgus subgroup (between 11- 23% and 30–92% of the stance phase), and the uncorrectable varus subgroup compared to the valgus subgroup (between 14-18% and 22–88% of the stance phase).

In the anterior-posterior direction, the center of pressure trajectory was significantly different between (sub)groups between 1-38% and 48–95% of the stance phase (F(4,1251) = 4.0, p < 0.001). The center of pressure trajectory was significantly more anterior for all HMSN subgroups compared to healthy controls during the first part of the stance phase (neutral: between 1-36% of the stance phase, correctable varus: between 1-35% of the stance phase, uncorrectable varus: between 1-33% of the stance phase, valgus: between 1-31% of the stance phase). The center of pressure trajectory was significantly more posterior for all HMSN subgroups compared to healthy controls during terminal stance (neutral: between 55- 83% of the stance phase, correctable varus: between 48-94% of the stance phase, uncorrectable varus: between 55-89% of the stance phase, valgus: between 60-85% of the stance phase) Additionally, the anterior-posterior center of pressure trajectory was significantly more posterior for the correctable varus subgroup compared to the neutral subgroup (between 84-87% of the stance phase) and for the correctable varus compared to the valgus subgroup (between 90-91% of the stance phase).

Mean center of pressure velocity values were significantly different between (sub)groups (F(4,1251) = 157.9, p < 0.001; healthy controls: 0.34 ± 0.05 m/s; neutral: 0.19 ± 0.10 m/s; correctable varus: 0.22 ± 0.09 m/s; uncorrectable varus: 0.18 ± 0.09 m/s; valgus: 0.19 ± 0.10 m/s). Post hoc tests revealed significant differences between healthy controls and all HMSN subgroups. No significant differences were found between the HMSN subgroups.

## Discussion

Larger root mean square deviations were found for all four foot deformity categories compared to healthy controls, indicating abnormalities in the plantar pressure patterns of people with HMSN. Detailed evaluation of the complete plantar pressure patterns revealed decreased plantar pressure underneath the rearfoot in the case of a plantar flexed position of the first metatarsal, increased plantar pressure underneath the lateral side of the foot in the case of a correctable or uncorrectable hindfoot varus, and decreased plantar pressure underneath the distal part of the second/third metatarsals in all foot deformity categories. The fifth metatarsal head pressure ratio was the best discriminant plantar pressure ratio to differentiate between healthy controls and people with HMSN and between the four foot deformity categories. Center of pressure trajectories were more lateral throughout the stance phase in the case of a correctable or uncorrectable hindfoot varus compared to healthy controls. In the anterior-posterior direction, the center of pressure trajectory was more anterior during loading response and midstance and more posterior during terminal stance for all HMSN subgroups compared to healthy controls. Hence, this study revealed spatially and temporally distinct plantar pressure patterns for each of the four foot deformity categories in people with HMSN.

The current study provided a deeper insight into the plantar pressure patterns of people with HMSN by evaluating plantar pressure patterns for each of the four foot deformity categories separately. Although clear differences in plantar pressure pattern were seen between the HMSN subgroups, all subgroups showed an increased pressure underneath the midfoot, which is in line with previous findings [5–8]. Prior studies that evaluated pressure underneath the rearfoot showed inconsistent results [5–8], while our study revealed decreased pressure underneath the rearfoot in people with HMSN. This discrepancy could possibly be explained by differences in foot deformity since previous research did not clearly distinguish between different foot deformity categories in the analysis of the plantar pressure measurements [5–7]. Furthermore, the pressures in the previous studies were not normalized for total pressure [5, 6, 8] and were, therefore, highly influenced by body weight.

As a general measure of abnormality, we introduced the RMSD of a plantar pressure pattern from the mean plantar pressure pattern of healthy controls. The RMSD increased with increased severity of foot deformity, i.e. the lowest RMSD was found for healthy controls, followed by the neutral subgroup, the valgus subgroup, and the correctable varus subgroup, while the highest RMSD was found for the uncorrectable varus subgroup. Therefore, the RMSD seems to be a powerful outcome measure to identify abnormalities in the plantar pressure patterns of people with HMSN. While the RMSD provides an overall view of the severity of a foot deformity, the plantar pressure ratios provide insight into the location of the abnormalities. The fifth metatarsal head pressure ratio appeared to be a strong discriminant to differentiate between healthy controls and people with HMSN and between the foot deformity categories. This ratio is especially capable of capturing pressure abnormalities of foot deformities with a varus position of the hindfoot since the fifth metatarsal head pressure ratio was significantly different between healthy controls, the neutral subgroup, the correctable varus subgroup, and the uncorrectable varus subgroup. Hence, we propose to use the RMSD, as a measure of overall abnormality of a plantar pressure pattern, in combination with the fifth metatarsal head pressure ratio, as a measure of varus deformity of the hindfoot, as outcome measures to evaluate foot impairments in people with HMSN.

A varus deformity of the hindfoot caused a more lateral center of pressure trajectory throughout the stance phase, which is in line with previous research [17]. Furthermore, a more anterior foot landing and a reduction in the forward progression of the tibia resulted in differences in the anterior-posterior center of pressure trajectory between healthy controls and all HMSN subgroups. However, only a few differences were found between the HMSN subgroups. Furthermore, in the anterior-posterior direction, it is difficult to interpret the results since it is unknown whether the reduction in forward progression of the tibia is caused by foot deformities (pes equinus) or by a compensation strategy for calf muscle weakness. Moreover, in the medio-lateral direction, a shift in the center of pressure trajectory between (sub)groups was found, but the course of the center of pressure trajectory over the stance phase was similar for the (sub)groups, indicating that the temporal investigation of plantar pressure seem to be less crucial for the evaluation of foot deformities in this population. Hence, center of pressure trajectories seem to be a less valuable outcome measure for the evaluation of foot deformities in people with HMSN.

Future research to establish the responsiveness of the proposed outcome measures to surgical interventions is recommended. Plantar pressure measurements have been used before as outcome measures for surgical interventions in people with HMSN and foot deformities [7, 17–19]. However, in most studies the number of foot areas that were studied was limited to five: the rearfoot, lateral and medial midfoot, and lateral and medial forefoot [7, 18, 19]. This makes it impossible to identify differences in the plantar pressure distribution underneath the distal part of the metatarsals, whereas the current study highlights the importance of studying the plantar pressure underneath the metatarsal heads separately for the evaluation of foot impairments in people with HMSN. Therefore, the proposed outcome measures could improve the evaluation of surgical interventions in people with HMSN.

Center of pressure velocity, which is an indicator of walking velocity [13], was significantly lower for the HMSN subgroups compared to healthy controls. Previous research found decreased plantar pressure underneath the heel and medial part of the forefoot and increased pressure underneath the midfoot and lateral part of the forefoot with decreasing walking velocity [20, 21]. This suggests that the differences in plantar pressure distribution as found in the current study can partially be explained by differences in walking velocity between the (sub)groups. However, the studies described above investigated the effect of walking velocity on plantar pressure patterns within subjects, whereas the influence of walking velocity on plantar pressure patterns between subjects has shown to be limited [10]. Moreover, center of pressure velocity was not significantly different between the HMSN subgroups, while clear differences in plantar pressure distribution were found between these subgroups.

A limitation of our analysis method is that it requires information on the complete foot shape to be able to normalize the plantar pressure patterns and to define the foot areas. As a consequence, seven feet had to be excluded from analysis because of incomplete footprints due to severe equinus deformities. However, it is expected that without the exclusion of these severe foot deformities the differences in plantar pressure patterns between (sub)groups would have been even larger. Additionally, the number of subjects in the uncorrectable varus and valgus subgroups were limited because these foot deformities are less common in people with HMSN compared to the other foot deformity categories. Furthermore, each foot was analyzed separately in this study because of differences in foot deformity between the feet of a person, which was the case in 14 people that were included. This is considered a limitation of this study since the plantar pressure pattern of one foot is influenced by the other foot. Another limitation of this study is the classification of the foot deformities based on clinical examination. This clinical classification does not allow the foot impairments to be studied on a continuous scale, while the plantar pressure measurements do. Therefore, in future studies, it would be interesting to investigate whether plantar pressure ratios and RMSD values could be used as continuous variables to examine foot impairments.

## Conclusions

People with HMSN have spatially and temporally distinct plantar pressure patterns compared to healthy controls. Furthermore, differences in plantar pressure pattern were found between four foot deformity categories in people with HMSN. Root mean square deviation and the fifth metatarsal head pressure ratio are proposed as measures for overall abnormality of a plantar pressure pattern and varus deformity of the hindfoot, respectively. Therefore, these measures could be considered in future research as outcome measures for the scientific evaluation of surgical interventions.

## Data Availability

Data is not publicly available since participants did not give permission.

## Declarations

For this database study, ethical approval was not required. The study was conducted in accordance with the local ethical regulations.

## Abbreviations

HMSN: hereditary motor and sensory neuropathies
RMSD: root mean square deviation
MT1: first metatarsal
ROM: range of motion

## References

[1] Pareyson D, Marchesi C (2009). Diagnosis, natural history, and management of Charcot–Marie–Tooth disease. Lancet Neurol.

[2] Reilly MM, Murphy SM, Laura M (2011). Charcot-Marie‐Tooth disease. J Peripheral Nerv Syst.

[3] Louwerens JWK (2018). Operative treatment algorithm for foot deformities in Charcot-Marie-Tooth disease. Operative Orthopädie und Traumatologie.

[4] Nonnekes J, Hofstad C, de Greef-Rotteveel A, van der Wielen H, van Gelder JH, Plaass C (2021). Management of gait impairments in people with Charcot-Marie-Tooth disease: a treatment algorithm. J Rehabil Med.

[5] Cardoso J, Alves de Baptista CR, Sartor CD, Nascimento Elias AH, Marques Júnior W, Martinez EZ (2021). Dynamic plantar pressure patterns in children and adolescents with Charcot-Marie-Tooth disease. Gait Posture.

[6] Burns J, Crosbie J, Hunt A, Ouvrier R (2005). The effect of pes cavus on foot pain and plantar pressure. Clin Biomech Elsevier Ltd.

[7] Chan G, Sampath J, Miller F, Riddle EC, Nagai MK, Kumar SJ (2007). The role of the dynamic pedobarograph in assessing treatment of cavovarus feet in children with Charcot-Marie-Tooth disease. J Pediatr Orthop.

[8] Crosbie J, Burns J, Ouvrier RA (2008). Pressure characteristics in painful pes cavus feet resulting from Charcot–Marie–Tooth disease. Gait Posture.

[9] Whitaker JM, Rousseau L, Williams T, Rowan RA, Hartwig WC (2002). Scoring system for estimating age in the foot skeleton. Am J Phys Anthropol.

[10] Keijsers NLW. Plantar pressure analysis. The science of footwear. CRC press; 2012. pp. 377–408.

[11] Garbalosa JC, McClure MH, Catlin PA, Wooden M (1994). The frontal plane relationship of the forefoot to the rearfoot in an asymptomatic population. J Orthop Sports Phys Therapy.

[12] Keijsers NLW, Stolwijk NM, Nienhuis B, Duysens J (2009). A new method to normalize plantar pressure measurements for foot size and foot progression angle. J Biomech.

[13] Keijsers NLW, Stolwijk NM, Renzenbrink GJ, Duysens J (2016). Prediction of walking speed using single stance force or pressure measurements in healthy subjects. Gait Posture.

[14] Maris E, Oostenveld R (2007). Nonparametric statistical testing of EEG-and MEG-data. J Neurosci Methods.

[15] Keijsers NLW, Stolwijk NM, Louwerens JWK, Duysens J (2013). Classification of forefoot pain based on plantar pressure measurements. Clin Biomech Elsevier Ltd.

[16] Pataky TC, Vanrenterghem J, Robinson MA (2015). Zero-vs. one-dimensional, parametric vs. non-parametric, and confidence interval vs. hypothesis testing procedures in one-dimensional biomechanical trajectory analysis. J Biomech.

[17] Metaxiotis D, Accles W, Pappas A, Doederlein L (2000). Dynamic pedobarography (DPB) in operative management of cavovarus foot deformity. Foot Ankle Int.

[18] Erickson S, Hosseinzadeh P, Iwinski HJ, Muchow RC, Talwalkar VR, Walker JL (2015). Dynamic pedobarography and radiographic evaluation of surgically treated cavovarus foot deformity in children with Charcot–Marie–Tooth disease. J Pediatr Orthop B.

[19] Lin T, Gibbons P, Mudge AJ, Cornett KMD, Menezes MP, Burns J (2019). Surgical outcomes of cavovarus foot deformity in children with Charcot-Marie-Tooth disease. Neuromuscul Disord.

[20] Rosenbaum D, Hautmann S, Gold M, Claes L (1994). Effects of walking speed on plantar pressure patterns and hindfoot angular motion. Gait Posture.

[21] Burnfield JM, Few CD, Mohamed OS, Perry J (2004). The influence of walking speed and footwear on plantar pressures in older adults. Clin Biomech Elsevier Ltd.

